# Chemical-Based Methodologies to Extend the Shelf Life of Fresh Fish—A Review

**DOI:** 10.3390/foods10102300

**Published:** 2021-09-28

**Authors:** Renata A. Amaral, Carlos A. Pinto, Vasco Lima, Jéssica Tavares, Ana P. Martins, Liliana G. Fidalgo, Ana M. Silva, Maria M. Gil, Paula Teixeira, Joana Barbosa, Francisco J. Barba, Jorge A. Saraiva

**Affiliations:** 1 LAQV-REQUIMTE, Department of Chemistry, University of Aveiro, 3810-193 Aveiro, Portugal; renata.amaral@ua.pt (R.A.A.); carlospinto@ua.pt (C.A.P.); vasco.lima@ua.pt (V.L.); jessica.tavares19@ua.pt (J.T.); ana.patricia.martins@ua.pt (A.P.M.); liliana.fidalgo@ipbeja.pt (L.G.F.); 2 Department of Applied Technologies and Sciences, School of Agriculture, Polytechnic Institute of Beja, 7800-295 Beja, Portugal; 3 Sonae MC, Rua João Mendonça 529, 4464-501 Senhora da Hora, Portugal; AMSILVA@sonaemc.com; 4 MARE—Marine and Environmental Sciences Centre, ESTM, Polytechnic of Leiria, 2520-620 Peniche, Portugal; maria.m.gil@ipleiria.pt; 5 Centro de Biotecnologia e Química Fina—Laboratório Associado (CBQF), Escola Superior de Biotecnologia, Universidade Católica Portuguesa, Rua Diogo Botelho 1327, 4169-005 Porto, Portugal; pcteixeira@porto.ucp.pt (P.T.); jbarbosa@porto.ucp.pt (J.B.); 6 Nutrition and Food Science Area, Preventive Medicine and Public Health, Food Science, Toxicology and Forensic Medicine Department, Faculty of Pharmacy, Universitat de València, Avda, Vicent Andrés Estellés, s/n, Burjassot, 46100 València, Spain; francisco.barba@uv.es

**Keywords:** fresh fish, spoilage, organic acids, natural extracts, vacuum packaging, modified atmosphere packaging, active packaging

## Abstract

Due to its characteristics, fresh fish is a highly perishable food with a very short shelf-life under refrigeration. Several methods have been introduced to slow down its deterioration, such as by means of oxygen depletion of the food package (vacuum packaging), or by changing the natural atmosphere that is in contact with the fresh fish (modified atmosphere packaging), or by the use of chemicals generally recognized as safe: such compounds can be directly applied (by dipping or spraying) or incorporated into packaging materials and slowly migrate to the product, exerting a hurdle effect against microbial development and lipid oxidation (active packaging). This review aims to cover the most recent advances in chemical-based approaches for fresh fish preservation, applied either singly or in combination. Vacuum packaging, modified atmosphere, and active packaging preservation methodologies are presented, along with the inclusion of chemical additives, such as organic acids and natural extracts, and their combination with icing systems. Advantages and disadvantages of these methodologies and their impact on fresh fish quality and shelf-life are discussed, reaching the conclusion that both are positively influenced overall. Indeed, the contribution of chemical-based strategies for fresh fish preservation is undeniable, and is expected to be a research topic of increasing interest in the future.

## 1. Introduction

Fish is a highly consumed and nutritious food with a high protein and low saturated fat content, and is a source of high quality polyunsaturated fatty acids (PUFAs), such as omega-3 (DHA—docosahexaenoic acid and EPA—eicosapentaenoic acid, for example) that are truly relevant for cardiovascular disease prevention and exhibit anti-inflammatory properties [1,2]. Due to their nutritional characteristics and composition, fish products are highly perishable foods. Their degradation, coupled with economic pressures, results in large quantities of product waste which, even with increased public awareness and investment from organizations, leads to over 20% of the annual production of fish products not being consumed [3].

Fish is increasing in popularity mostly as a fresh product due to the convenience for subsequent preparation and cooking, but it can easily deteriorate due to its elevated water content, postmortem pH, and the presence of small molecules (free amino acids) which make it extremely vulnerable to bacterial and chemical spoilage.

Fish spoilage combines different processes such as enzymatic autolysis, oxidation, and microbial growth and it depends on intrinsic characteristics such as the activity of endogenous enzymes, initial bacterial composition, muscle tissue fragility and extrinsic factors related to water quality, aquaculture practices, food handling and packaging, storage, and transportation conditions, among others. These lead to changes in odor, flavor, and texture mainly because of drip loss, discoloration, protein degradation, nucleotide decomposition, accumulation of nitrogenous compounds, and lipid and protein oxidation [2,4]. Furthermore, besides spoilage bacteria, the growth of pathogenic microorganisms is a cause of concern. Fish-associated foodborne illnesses have been linked to a variety of viruses, bacteria, and parasites [5].

Some chemical quality indicators are used to assess the extension of fish spoilage, such as the total volatile basic-nitrogen (TVB-N) and trimethylamine-nitrogen (TMA-N), thiobarbituric acid (TBA) value, and the presence of biogenic amines (histamine, cadaverine, tyramine and putrescine) produced by the decarboxylation of specific free amino acids by the action of microorganisms [6,7]. TVB-N includes the measurement of volatile basic nitrogenous compounds such as trimethylamine (TMA), dimethylamine (DMA), and ammonia (NH_3_) which are produced by bacteria, from the action of enzymes, or from the deamination of amino acids [8,9,10]. The proposed value of TVB-N for spoilage initiation is 30–35 mg N/100 g; however, some studies present lower levels depending on the fish species [9,10,11]. TMA-N is produced by bacterial spoilage and enzymatic activity and the decomposition of TMA-N-oxide (major constituent of non-protein nitrogen fraction) is responsible for the fishy odor. Values of 10–15 mg TMA-N/100 g are considered the upper limit for spoilage acceptance, but lower limits are also suggested by other authors [8,9,10]. Regarding lipid oxidation, it is the TBA value that measures the malondialdehyde (MDA) content, which is formed by the reaction with hydroperoxides. Quality values range between 2–4 mg MDA/kg, but this value might not reflect the actual rate of lipid oxidation because MDA can interact with other components [9].

Fish is usually stored under refrigeration, presenting a very short shelf-life, or under freezing conditions, exhibiting a longer shelf-life but losing important quality properties [12]. In fact, storage under −18 °C is the most effective and common method used, but this preservation technique leads to ice crystals formation that damages the muscle structure. In addition, thawing prior to cooking is a time-consuming process [4].

For this reason, the development of innovative approaches for shelf-life extension became inevitable, always with the goal of achieving the best sensorial and nutritional quality and safety.

Fish can also be packaged under vacuum or under modified atmosphere, as these food packaging methodologies have been used to preserve the quality of foods, slowing down its deterioration until consumption. Packaging is used for protection from the external environment, communication, convenience for an easier handling of the product, and containment for different sized and shaped products to optimize the logistic process [13]. Both vacuum-packaging (VP) and modified atmosphere packaging (MAP) have essentially remained unchanged since they were introduced, at the beginning of the last century, and are well-established in the food industry. Nevertheless, increasing concerns regarding food quality control and preservation have led to a new role for packages: the extension of shelf-life [13,14].

Several preservation strategies have been evaluated, considering a minimal impact on fish texture. These strategies rely on the application of additional hurdles prior to refrigerated storage, such as natural extracts (with antimicrobial and/or antioxidant properties) or active packages, to reduce microbial loads to acceptable levels and reduce degradation reactions to increase the shelf-life of fresh fish. Chemicals such as organic acids or ozone are, by far, the most frequently used hurdles to inhibit microbial growth in foods [15]. However, they are being replaced by alternatives perceived as more natural, such as extracts from plants and algae, or bacteriocins from bacteria.

Nowadays, there are more advanced food packaging techniques available such as active and interactive and smart/intelligent packaging, characterized by the interaction between the packaging system and the food product, which react to biochemical changes in food andcan monitor the quality of products, proving visual information to the consumer about the products quality state, for example through a color system [16,17]. Therefore, the new food packaging is characterized by the interaction between the food and the internal packaging environment [16], while traditional packaging is characterized by an inert physical barrier between the food and the external environment.

This review will cover the most recent advances in chemical-based approaches for fresh fish preservation, either singly or simultaneously combined, comprising the main effects of each methodology on microbiological and chemical aspects of fresh fish.

## 2. Chemical Methodologies for Shelf-Life Extension

Nowadays, the shelf-life, food safety, quality, and nutritional composition of foods are the most important parameters for consumers, who are looking for increasingly natural, sustainable, fresh, and tasty foods [18]. VP and MAP technologies are two of the most well-established preservation technologies for fishery products and are characterized by the removal of oxygen in order to slowdown microbial proliferation and oxidative reactions [12,18,19]. Active packaging (AP) is also a packaging technology, but it interacts directly with food, such as fresh fish, in order to preserve the quality and extend its shelf-life, instead of being just an inert barrier that separates it from the external environment. Additionally, it is important to note that these preservation methodologies still need chilled storage.

### 2.1. Conventional Packaging: Vacuum-Packaging and Modified Atmosphere Packaging

The concept of VP consists of removing the air from a package containing the product and sealing it immediately. It separates the product from the external environment, limits the package volume and prevents oxidative spoilage if the films used have oxygen (O_2_) barriers [19]. For these reasons, this low-cost technique is commonly used in the food industry [20].

The MAP process starts with the removal of the gaseous atmosphere present in the package and then inserting a mixture of selected gases before sealing the package. Typically, this mixture of gases has high levels of carbon dioxide (CO_2_), because of its antimicrobial effect inhibiting the growth of aerobic microorganisms [19,21]. This happens because CO_2_ dissolves in water and produces carbonic acid and a reduction of pH occurs. This leads to a prolongation on the lag phase and microbial growth is lowered throughout the logarithmic phase. However, the drop in pH could be minimal, and no bacteriostatic effect is noticed [22]. Nevertheless, CO_2_ can be prejudicial to sensorial acceptance, affecting cellular structures from the fish, as well all color and flavor [23]. Oxygen (O_2_) and nitrogen (N_2_) are also used, since the former inhibits strictly anaerobic bacteria and the latter delays oxidative rancidity and limits aerobic microorganism’s growth when used to replace oxygen on the package [9,21]. Ultimately, a wise combination of the abovementioned gases will result in an extension of shelf-life maintaining product quality, while it is also possible to design specific atmospheres to inhibit targeted and undesired microorganisms [21,24].

**Figure 1:**
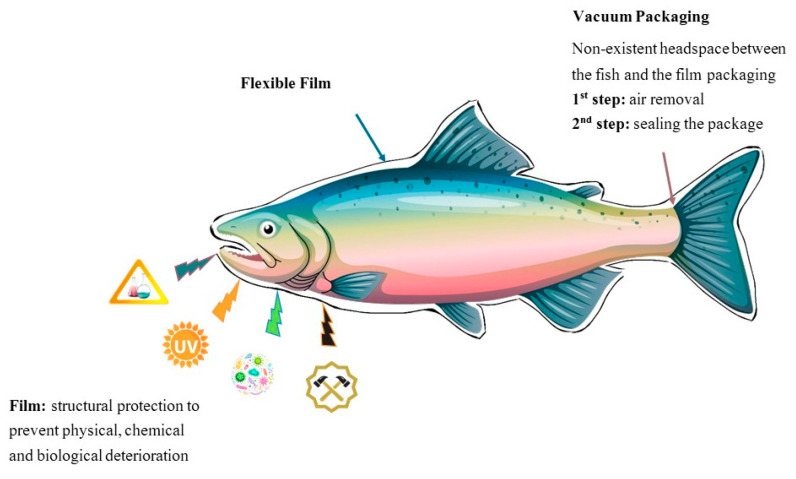
Schematic representation of VP technology applied to fishery products.

**Figure 2:**
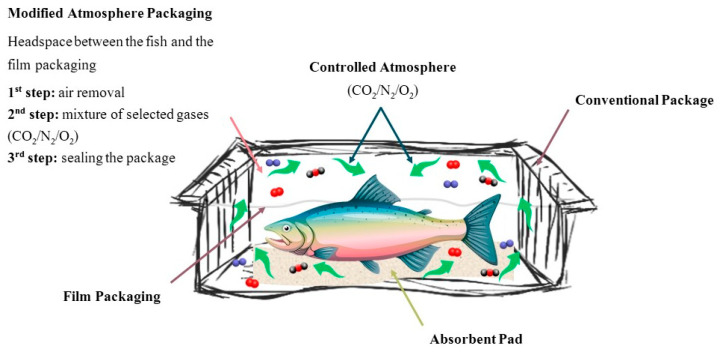
Schematic representation of MAP technology applied to fishery products.

It is important to highlight that regardless the type of packaging, fish preservation will be highly dependent on multiple factors, like type and number of microorganisms present in the water of the fish’s habitat, storage and transportation temperature, and handling during processing and distribution [25,26]. Furthermore, different fish species and experimental designs (type of packaging, ratio of gases, storage temperature) are studied and this could explain the differences observed on the results discussed below. Figure 1 and Figure 2 represent the mechanism of VP and MAP technologies, respectively.

The most important parameter for shelf-life evaluation is the development of microorganisms during storage. This evolution will be highly dependent on original microorganisms and their tolerance to the specific gases used.

Even so, it has been observed that MAP can reduce *Pseudomonas* spp. counts and H_2_S-producing bacteria. Likewise, atmospheres with higher CO_2_ content are more efficient in reducing lactic acid bacteria (LAB) counts.

Besides microbiological safety, preserving good sensorial quality is particularly important for shelf-life evaluation. Some studies [6,8,23] reported changes in color, odor, and texture along the shelf-life period with VP and MAP. Nonetheless, a study reported no sensory changes observed for a 100% CO_2_ atmosphere in common carp (*Cyprinus carpio*) [27].

Since VP and MAP affect microbial spoilage and the sensorial quality of foods, shelf-life will also be affected. Longer shelf-lives are usually observed with MAP, followed by VP and, lastly, air packages. Normally, VP preserves the quality of the product for 2/3 days longer, compared to air packaging [11,21,25,28]. With MAP, there tends to be an increase in shelf-life of 4/5 days compared to air packs and 2/3 days compared to VP [10,11,19,20,21,23,26]. However, some studies have reported different results (see more details in Table 1). It is important to note that temperature of storage and the atmosphere used are important factors that affect shelf-life. For example, Stamatis & Arkoudelos (2007a) [28] studied chub mackerel (*Scomber colias japonicus*) under air packaging, VP, and MAP (50% CO_2_ and 50% N_2_) at 3 and 6 °C and observed, as expected, that the lower temperature allowed for an extension of shelf-life of 2 days for MAP and VP samples and 1 day for air samples comparing to the higher temperature. One work with sutchi catfish (*(Pangasius hypophthalmus*) investigated two different atmospheres: (1) 50% CO_2_ and 50% N_2_; and (2) 50% CO_2_ and 50% O_2_, at 4 °C for 21 days and concluded that the second atmosphere, where O_2_ was used instead of N_2_, extended the shelf-life by 2 days because of an extension of lag phase (of microbial development) with this atmosphere [25]. Another study using sea bass (*Dicentrarchus labrax*) with two different MAP mixtures: (1) 40% CO_2_, 50% N_2_ and 10% O_2_; (2) 60% CO_2_, 30% N_2_ and 10% O_2_, at 4 °C during 21 days of storage, noticed an increase of shelf-life of about 4 or 5 days for the MAP samples with higher levels of CO_2_ [9]. Notwithstanding, Lerfall et al. (2018) [24] studied the effects of four different MAP mixtures in saithe (see the details in Table 1) and observed the same shelf-life for all of them.

The spoilage of fresh fish is not exclusively determined by microbial development, but also by the formation of chemical indicators that reflect, for example, autolytic changes in the fish muscle, formation of biogenic amines, among other rancidity and oxidation associated compounds, these indicators need to be considered based on the shelf-life evaluation of fresh fish [35], although shelf life is usually limited at first instance by microbial development. Indeed, biogenic amines were evaluated in fish samples stored under different packaging conditions. Briefly and additionally, major results found in the literature regarding VP and MAP are displayed in Table 1.

### 2.2. Active Packaging

Active packaging systems are an innovative and alternative solution of high interest to food technologists. Food packaging and materials are categorized as active, according to the Regulation of the Commission of the European Union (EC No 450/2009), when their application aims to extend the shelf-life and maintain or improve safety and some physicochemical, quality, and sensorial properties of the packaged food due to the positive interaction between product, package, and environment [16,17,36].

Therefore, active packaging is more than just an inert barrier that protects food from external detrimental factors. In fact, this technology is based on the incorporation of active substances, components, and materials into the packaging material or within the package, designed to release or absorb substances into or from packaged food or its surrounding environment [36,37,38,39]. It is noteworthy that the main difference between active and intelligent packaging systems is the information about modifications in the quality status of the food product that is communicated to the consumer using intelligent packaging, instead of interacting with it and responding to changes in its properties, as happens with active packaging [17]. Intelligent packaging only monitors the microbiological and physicochemical modifications (like biological reactions, pH, or temperature) of packaged foods or the surrounding environment, transmitting the information to the consumer, using devices such as sensors (including biosensors, gas sensors, printed electronics, chemical sensors, and electronic nose) and indicators (including freshness indicators, leak/integrity indicators and time and temperature indicators) [16,17]. Intelligent packaging is not designed to interact with the packaged food or release active compounds as in active packaging, it merely helps to detect unsafe food [16,17].

**Figure 3:**
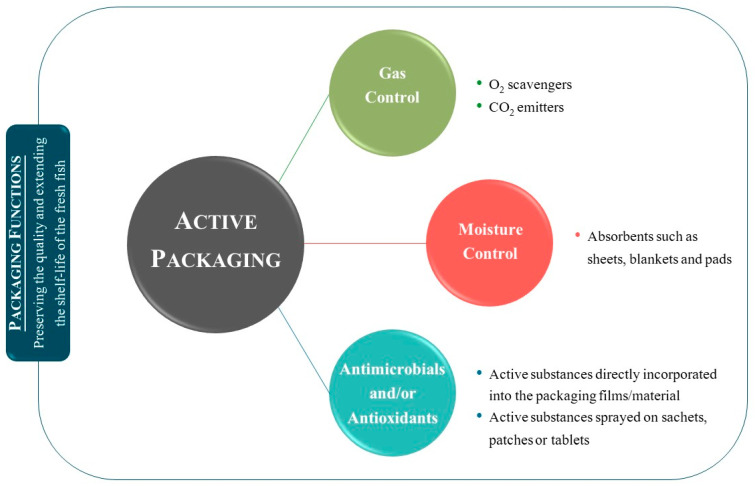
Active packaging technology applied to fishery products. Based on information available in [17].

This section is mainly focused on active packaging systems. The three main types and the most common and promising of active packaging systems of foods are: (i) gas control, including O_2_ scavengers and CO_2_ emitters, (ii) moisture control, and (iii) antimicrobials and/or antioxidants, which are active substances directly incorporated into the packaging material/films or sprayed on sachets, patches, or tablets [17,39]. To reduce, inhibit, or delay the microbial growth and enzymatic/oxidative reactions of food is the primary and common goal of all three methodologies [4]. Figure 3 summarizes the three main types of active packaging systems described above.

The gas control mechanism in active packaging technology is very similar and directly related to MAP. The substances that absorb (O_2_ scavengers) or release (CO_2_ emitters) atmospheric gases control the inside packaging environment. Briefly, O_2_ scavengers prevent the oxidation of lipids and other sensitive compounds (like vitamins A, C, and E) and, thus, off-flavors and off-odors; and, whereas the CO_2_ emitters slowdown or inhibit microbial growth. Nowadays, oxygen scavengers are the most used active packaging strategies and, as Remya et al. (2017) [40] reported, the commercial ZPT 200 EC O_2_ absorber (Ageless^®®^), when applied to fresh *Rachycentron canadum* steaks during refrigerated storge, enhanced fish quality and shelf-life (shelf-life of 25 days compared to just 15 days for control samples). In fact, this O_2_ scavenger reduced the O_2_ concentration in the package to less than 0.01%, which translated into inhibition of the growth of (i) aerobic mesophilic and psychrotrophic microorganisms due to the lag phase extension and generation time, (ii) *Pseudomonas* sp. because they are strictly aerobic, and (iii) H_2_S-producing bacteria, as their enzymes were affected by O_2_ depletion (effects observed when O_2_ concentration is below 50%). So, this oxygen-reduced atmosphere promoted the growth of Gram-positive microorganisms such as LAB (which are facultative anaerobes) and significantly affected the growth of Gram-negative bacteria in fish samples. Therefore, volatile base formation values (TVB-N and TMA-N, highly dependent on microbial load) were delayed, and a similar inhibitory effect was observed for lipid oxidation.

Moisture control is mediated by diverse kinds of absorbents (made from a micro porous polymer like polypropylene—PP—or polyethylene terephthalate—PET) such as sheets, blankets, and pads, which are strategically placed under food products (mostly fish and meat) to control their exudates [17,41]. Dri-Loc^®®^ (Sealed Air Corporation, Charlotte, NC 28208 USA) and Tenderpac^®®^ (SEALPAC, Oldenburg, Germany) are two commercially available moisture control devices based on an absorbent pad and a dual compartment system, respectively. In fact, this type of moisture absorber is widely used in the food industry to decrease water activity (an important intrinsic factor) in food and thus inhibit microbial spoilage [16].

There are two main methodologies for manufacturing antimicrobials and/or antioxidant packaging systems, which are (i) the incorporation of active compounds/substances into sachets, pads, or tablets (independent devices) that are added to the conventional inert package, and (ii) the direct incorporation of active compounds/substances in the polymer matrix or on the polymeric film surface [17,38].

**Figure 4:**
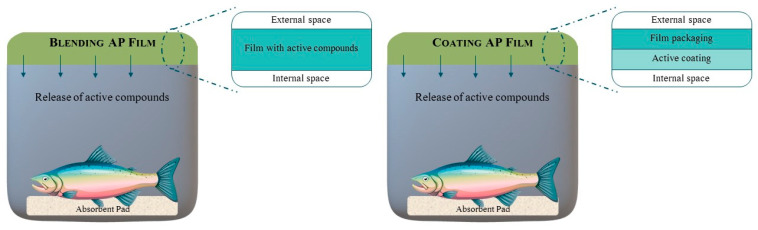
The graphic representations of blending and coating active packaging (AP) films applied to fishery products. Based on information available in [4].

Blending and coating packaging films are produced differently (as represented in Figure 4). While, with the first, the active compounds are combined with the polymeric matrix and, in the second they are coated onto the surface of the polymeric film [4,38]. These systems are defined by a controlled release (to the headspace of the package) or reaction of the active compounds onto the food surface [39].

To select the most suitable packaging film manufacturing procedure, it is important to consider the type of polymeric matrix, the active substances to be incorporated and the physicochemical characteristics of the food product [38].

The packaging films can be from synthetic or natural origin. The most used synthetic polymers are low-density polyethylene (LDPE) and polystyrene, due to their physical properties and low production cost, but their widespread use is cause for environmental concern, due to their low degradability. Barbosa-Pereira et al. (2013) [42] performed a 21-day experiment with salmon muscle, wherein the authors combined low-density polyethylene polymers (LDPE, a synthetic film) with two different formulations in tocopherols, namely natural antioxidant products C: NUTRABIOL^®®^—T90 at 1 and 5% and D: TOCOBIOL^®®^—PV at 5%. The active films with natural antioxidant products C and D showed an important reduction in lipid oxidation of up to 40%. Considering the Film LDPE product C at 1% (film 2) and 5% (film 3), there were some small differences between them in the first 11 days of storage, but at the end of the experiments the reduction in lipid oxidation stabilized around 40%. At 21 days of storage, Film 4 (D: TOCOBIOL^®®^—PV at 5%) was able to decrease lipid oxidation in salmon by 30-35%; however, the greatest inhibition of lipid oxidation was observed for film 3, possibly due to its tocopherol composition. Therefore, it can be concluded that the inhibition of the lipid oxidation process is directly correlated with the capacity of active compounds (present in commercial antioxidant products C and D) to scavenge free radicals and peroxide radicals from the peroxidation chain reaction.

Thus, biopolymers—composed mainly of proteins and polysaccharides or their combinations (such as chitosan, agar, and starch films)—have gained more attention for being extracted from natural and renewable sources, which gives them some advantages, such as specific biological activities (antimicrobial and/or antioxidant activity, for example) and the possibility of being renewable. Natural active compounds with antimicrobial and/or antioxidant capacities can be found in extracts from spices (like rosemary, cinnamon, and oregano), herbs (such as garlic, onion, and horseradish), and from food processing by-products (for example, olive leaves and pomegranate peel), essential oils, fungal and bacterial compounds (polypeptides like nisin, natamycin, and pediocin, and some bacteriocins), and functional enzymes. These compounds can be incorporated into packaging films, as opposed to synthetic ones (like chlorine dioxide, carbon dioxide and ethanol, which are volatile antimicrobials) [4,16,17]. However, to enable the application of these natural films in food, it is necessary to add plasticizers to reduce their brittleness, and neutral lipids to increase their hydrophobicity [4,17].

There has been an increase in available literature featuring the combination of biopolymers and natural active compounds to formulate new films to be used as active packaging materials. Rocha et al. 2018 [43] carried out a detailed experiment to evaluate the antimicrobial effect of protein hydrolysate (PH, a by-product obtained from Argentine croaker—*Umbrina canosai*—which has a high protein content) and/or clove essential oil (CEO) combined with agar film in prolonging the shelf-life of flounder (*Paralichthys orbignyanus*) fillets. A 15-day assay at 5 °C, showed that the PH–agar film with lower molecular weight (<10 kDa) has a higher content of hydrophobic amino acids (~50.0%) which, due to their positive charge, allowed the formation of hydrophobic bounds with the negatively charged bacterial cell membrane, leading to its disruption and, proving to be an effective film against to H_2_S-producing microorganisms, lactic acid bacteria, and total aerobic mesophiles. CEO–agar film also extends the shelf-life of flounder fillets, in a slightly superior manner compared to PH–agar film, due to its recognized antimicrobial properties—its hydrophobicity allows the disruption of bacterial cell membrane lipids and mitochondria, leading to loss of cytoplasm, and, in addition, phenolic compounds can interact with bacterial enzymes, promoting lesions that lead to loss of cell viability. So, these CEO/PH–agar films performed well in extending the shelf-life of flounder fillets by microbial inhibition and enhanced their properties. This study also evaluated the mechanical properties of films such as thickness, tensile strength (TS), elongation break (EB), water vapor permeability (WVP), solubility, color, opacity, and transparency, which are very important parameters for evaluating the applicability of these active films in foods. The PH–agar film showed the best properties when compared to the CEO-agar film, with similar thickness and transparency to the control–agar film, but with an increase in WVP and EB values and a reduction in the TS values, due to the presence of short chain hydrolysate peptides that act as effective plasticizers in the protein films, by decreasing intramolecular attractive forces and increasing chain mobility and free volume (EB and WVP parameters) and by preventing the polymer–polymer interactions (TS parameter). The main disadvantage of this PH–agar film is related to the greater water solubility due to the weak interaction between the short PH peptides and the agar matrix, which limits its application in fish preservation.

Regarding biopolymer enriched active compounds and their advantages for the environment compared to synthetic ones, advances in active packaging technology have been made to meet consumers’ demands for healthier, greener, and more sustainable packaging materials. In this context, Kakaei & Shahbazi (2016) [44], among many other studies, investigated the effect of chitosan–gelatin films enriched with ethanolic red grape seed extracts (GSE, at 1–2%) and *Ziziphora clinopodioides* essential oil (ZEO, at 1–2%), on minced rainbow trout fillet. After 11 days of storage experiments (at 4 °C) the control samples (wrapped only with chitosan–gelatin film) faced an increase of about 9.0 log CFU/g for total viable counts (TVC) and psychrotrophic total counts (PTC), with a higher microbial reduction verified for the film with the combination of 2% ZEO + 2% GSE, where TVC increased up to 6.5 log CFU/g and PTC reached about 6.0 log CFU/g. The same behavior was observed for the other microorganisms tested, namely *Pseudomonas* spp., *Pseudomonas fluorescens*, *Enterobacteriaceae*, LAB, and *Listeria monocytogenes* where the same combination of 2% ZEO + 2% GSE showed the lowest counts after 11 days of storage (ranging between ~3.5 and 5.0 log CFU/g), when compared to the other ZEO+GSE combinations. The microbial reduction for the 2% ZEO + 2% GSE films was even more relevant when compared to the control samples (on day 11 counts were in a range between ~7.0 and 8.0 log CFU/g for control samples). Therefore, these results showed the high effectiveness of the chitosan–gelatin films in retarding and inhibiting the growth of aerobic microorganisms, as they act as an oxygen barrier around the fish and additionally block access to essential nutrients and metals by bacteria. Furthermore, the presence of ZEO and GSE compounds in the films causes a significant antibacterial effect due to their high content of phenolic compounds (ZEO has mainly thymol and carvacrol and GSE has mainly resveratrol, gallic acid, caffeic acid, and hydroquinone), which, when combined, exert a synergistic antimicrobial effect, such as the inhibition of bacterial enzymes and the establishment of bonds with cell membrane components that promote the loss of cell viability.

Physicochemical analyses, namely of pH value, peroxide value (PV), and total volatile base nitrogen (TVB-N) content, were also performed and it was concluded that the films with 2% ZEO + 2% GSE obtained the best results. In the case of pH, the final pH value, at 11 days of storage, was 7.4 in contrast to ~6.7 for the best film, because of the bacterial reduction and due to decomposition of nitrogenous/alkaline compounds. Considering the PV values, the control samples exceeded the legal limit for fresh fish, showing a value of 1.9 meq peroxide/1000 g lipids (legal value for fresh fish is between 0.04–0.06 meq peroxide/1000 g lipids) at a higher rate when compared to active films samples. Finally, the TVB-N content values reinforce the effectiveness of chitosan–gelatin films with ZEO and GSE, because it was found that the TVB-N content of control samples significantly increases compared to the acceptability value of 25 mg N/100 g of fresh fish, in contrast with ZEO and GSE, where the values remained below the limit—the possible explanation being the lower microbial load or the low capacity of the remaining bacterial load to carry out the oxidative deamination reaction of the non-protein nitrogenous compounds.

Active packaging systems have been investigated to preserve perishable foods, namely fish, pork/bovine/poultry meat products and their derivatives, as well as for fruits, vegetables, and dried products, among others [4,16,38]. In fact, it is for fish and fishery products that this technique is most promising, due to its benefits for foods (safety, quality and shelf-life), industry, and consumers [4,39]. However, the adoption of active film packaging systems depends on factors such as production cost and legislative approval of active substances and polymer matrices for use in contact with food, even though they are useful to extend the shelf-life, improve safety, and improve or maintain quality and sensorial parameters of fish and fishery products [16,39].

Given the diversity of matrix polymers and active compounds, it is necessary to choose the most appropriate combination, based on the characteristics of fish and fishery products [4]. By adjusting the film system, the release rate of active compounds must be defined to retain a specific concentration in the fish-based products and to compensate for biochemical reactions during storage [4,37]. Examples of active packaging films where natural active compounds have been incorporated into natural/synthetic polymers in fish and fishery products are represented in Table 2.

There are already some commercially available antimicrobial packaging systems such as Biomaster^®®^ and Agion^®®^ for active packaging systems, but also O_2_ scavengers and moisture control devices [16].

### 2.3. Chemical Additives

The aforementioned preservation strategies relied on either changing the natural atmosphere wherein fresh fish was packaged or by the impregnation of packaging materials with chemicals that slowly migrate to fresh fish, exerting an antioxidant and/or antimicrobial effect. Nevertheless, such chemicals can be applied directly to fresh fish, either by dipping or spraying prior to packaging, providing an additional hurdle to the refrigeration processes.

**Figure 5:**
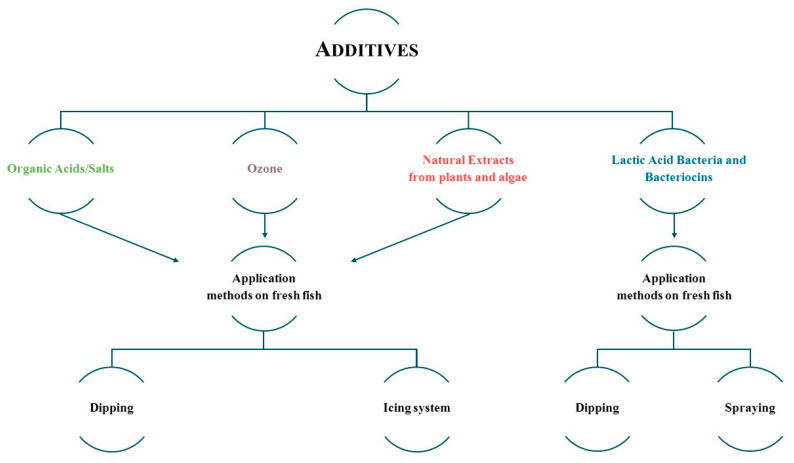
Chemical additives used to preserve fishery products and their application methods.

This section will focus on the main chemicals (single or in combination with icing systems) to preserve fresh fish, as well as natural extracts, bacteriocins, among others, which have different effects on fish quality and shelf-life. Figure 5 summarizes the main chemicals and application methods used to preserve fresh fish.

#### 2.3.1. Organic Acids

Organic acids are known to have antimicrobial and antioxidant properties, are generally recognized as safe (GRAS) [48], and their use as food preservatives has been extensively reviewed and forbidden in some cases, as is the case for benzoic acid, whose use is being gradually reduced/eliminated in several countries due to toxicity issues [49].

The organic acids action against microorganisms depends on the carbon chain length and degree of unsaturation, but overall, the pKa of the acid influences its antimicrobial mechanism of action [50]. The organic acids exist in a pH-dependent equilibrium between the undissociated and dissociated state, and the first one is the primarily responsible for the antimicrobial activity [51,52]. At low pH, they have an optimal inhibitory effect, which is a result of the undissociated organic acid being able to freely cross the plasma membrane to enter the bacterial or fungal cell. Once inside the cell, the molecule confronts a higher pH and will then dissociate, releasing charged anions and protons [52]. These accumulating anions have been found to be toxic and able to inhibit metabolic reactions [50]. Other mechanisms have been proposed, as the case of membranes disruption and stress on intracellular pH homeostasis [53,54]. According to the available literature, fresh fish preservation by organic acids/salts may follow two different strategies, namely by dipping fish samples for a certain period, followed by packaging and refrigeration, or by using organic acids to prepare flake ice for cooling fresh fish.

##### Fresh Fish Dipping

The first known report, as the authors are aware, regarding the use of a dipping process with organic acids in fresh fish belongs to Martinez & Gilderb (1988) [55], who showed the possibility of reducing anchovy (*Engradis encrasicholus*) degradation by pH depletion from 8 to 5, followed by cold storage at 1 °C in sea water for 35 h. The pH depletion allowed partial inhibition of the activity of proteolytic enzymes.

Some factors may be considered when applying the dipping strategy, such as the concentration of organic acid, dipping time, and air-availability within the package (air or vacuum package) as well as the storage temperature and draining time after dipping. For example, Kim, et al. (1995) [56] evaluated the effects of lactic acid (2 and 3%) dipping (1 and 5 min) on catfish fillets (*Silurus glanis*) and observed that, for the lowest lactic acid concentration, the dipping time influenced microbial development over 9 days of storage (at 4 and 10 °C), with microbial loads being quite similar for the concentration of 2% with 5 min of dipping time and 3% concentration with 1 min of dipping time.

Manju et al. (2008) [57] evaluated the effects of sodium acetate and potassium sorbate, both at a concentration of 2%, on pearl spot (*Etroplus suratensis*) dipped for 30 min and stored under refrigerated conditions (1–2 °C) in combination with vacuum- and air-packaging. The authors reported a shelf-life extension, in both packaging methods, of up to 16 days when compared to control samples (7 days without organic acid salts). The addition of sodium acetate and potassium sorbate yielded considerable reductions in TVB-N and TMA-N, improved textural properties, and slowed down microbial development, especially for vacuum-packed samples, which correlated well with the lower levels of TVB-N and TMA-N found in samples packed at such conditions, with the authors stating that this could be due to microbial development control, as well as the decrease of bacteria’s ability to perform oxidative deamination of non-protein nitrogen. In addition, the authors observed that samples treated with potassium sorbate presented lower values of TVB-N compared to those treated with sodium acetate, which was thought to be due to the greater inhibition of the first salt on Gram-negative bacteria. The susceptibility of Gram-negative bacteria to acidity was also noticed by Kim et al., (1995) [56], who stated that undissociated organic acids may penetrate within the lipid membranes of microorganisms, leading to the protonation of anionic components (as is the case for phosphate and carbonyl groups) of such structures, weakening molecular interactions, leading to membrane disruption [58]. Organic acids may also penetrate within microbial cytoplasm, where they usually dissociate and tend to donate protons (H^+^), forcing microorganisms to expel the excess of H^+^, which is an energy consuming process, thus limiting microbial proliferation. Consequently, the cytoplasm of the microorganisms reaches pH levels that are unbearable for microbial development [59].

This could be an interesting strategy to inhibit pathogens such as *L. monocytogenes*, *Escherichia coli* [60], and *Vibrio* spp. [61] and reduce bacterial loads responsible for fish spoilage, as is the case for *Pseudomonas* spp., LAB, H_2_S-producing bacteria, and *Enterobacteriaceae* [62]. These are the main bacterial groups responsible for fish spoilage and foodborne illnesses caused by the consumption of raw or undercooked fish. Additionally, pathogens such as *L. monocytogenes* and *E. coli* could also be inhibited [60].

While the dipping strategy seems to have quite beneficial effects on the shelf-life of fresh fish, high dipping times and organic acid concentrations may lead to fish muscle digestion and undesirable sensorial attributes, especially of color parameters (which can be minimized with the cooking process) [57]. Usually, during fish dipping, the fish pH tends to decrease, depending on the organic acid concentration and dipping time, which will have a major impact on fish texture, and in water-holding capacity (WHC), as when the pH of fish reaches the isoelectric point, proteins tend to aggregate or denature, leading to the reduction of WHC [63]. In addition, the pH of fish tends to increase during cold storage due to microbial deamination and protein breakdown, so dipping fish in organic acids can slow down microbial proliferation (as previously described) and inhibit some enzymatic reactions [64] given these effects on proteins. Some other examples of studies covering fresh fish dipping and spraying are displayed in Table 3. In addition to the effects of fish dipping previously described, one interesting effect of this technique is the reduction of heavy metals levels in fish from fresh and sea waters, for instance, a reduction of heavy metals was verified in freshwater fish, Tilapia nilotica (*Oreochromis niloticus*), pre-treated with acetic acid, which could be due to the formation of insoluble acetate salts of these metals [65]. Briefly, Elnimr (2011) observed a reduction of 41.6 and 51.9% of cadmium and lead in Basa fish (*Pangasius hypothalmus*) after dipping in acetic acid solution (5%) for 15 min.

##### Fresh Fish Organic Acid-Icing

The organic acid-icing process consists in preparing ice flakes from aqueous solutions of organic acids that will be directly in contact with the fish surface, thus allowing a slow migration of such compounds, as well the possibility of keeping fresh fish under chilling conditions near 0 °C.

In this sense, Rey et al. (2012) [75] assessed the performance of an icing-system containing ascorbic, citric, and lactic acids, with concentrations of about 400 and 800 mg/kg (C-400 and C-800) of ice under refrigeration conditions (4 °C) to preserve hake (*Merluccius merluccius*), megrim (*Lepidorhombus whiffiagonis*), and angler (*Lophius piscatorius*). Generally, it was verified that the C-800 icing-system retarded microbial development, along with lower levels of TMA-N formation for 12 days of storage, and with a good overall acceptance by the sensorial panel, which attributed a sensorial shelf-life between 8 (megrim) and 12 (hake and angler) days, especially concerning muscle odor and taste.

The effects of organic acid-icing (like the one described above) on fish lipid oxidation (primary, secondary, and tertiary) and hydrolysis were assessed by García-Soto et al. (2011) [76], using hake, megrim, and angler as case studies. The results showed that lipid oxidation was partially inhibited in the three studied fishes, especially for the icing-system containing the highest organic acid concentration (C-800). The authors attributed this effect to the antioxidant properties of the organic acids used. Lipid hydrolysis (free fatty acids content) was also generally inhibited, especially in megrim and angler, by the C-800 icing-system. The hydrolysis of lipids is, in a first phase, mainly due to endogenous enzymatic reactions carried out by lipases and phospholipases, and by microbial activity in a second phase. So, it is noteworthy that the pH of fish samples can be depleted by the icing system, resulting in partial enzymatic reaction inhibition (as explained before for fish dipping), as well as by inhibition of microbial development in lean fish. This behavior was validated in a fatty fish (mackerel, *Scomber scombrus*) by Sanjuás-Rey et al. (2012) [77], who observed microbial development inhibition and lower values of TMA-N and TVB-N by using an icing-system consisting of 0.05% of citric, acetic, and lactic acids.

#### 2.3.2. Ozone

The first report of ozone usage in the food sectors dates from 1936, when it was used in France to treat shellfish, and was recognized as a secondary direct food additive to destroy food pathogenic microorganisms in 2001 by the Food and Drug Administration (FDA). From this date onwards, several industries have been using ozone as a surface decontaminant, especially for fruits, vegetables, eggs, seafood, meat, sausage, and dairy industries [78].

The application of ozone to fresh fish follows a strategy of ozone dissolution in water, with the fish individuals or processed fish being dipped for a certain period, or it can be combined with icing systems, where it is stored at temperatures slightly below 0 °C, remaining in contact with the ozone (dissolved and entrapped in ice) during the storage period. In this section, both techniques will be presented and then discussed in the next section.

##### Ozonized Water Dipping

As previously stated, this strategy consists of dipping fresh fish in ozonized water for a certain period. Several variables are to be considered when performing this method, such as ozone concentration, exposure time (dipping time), and ozonized-water temperature.

When it comes to ozone concentration, it is expected that higher concentrations may yield higher microbial load inactivation/injuries, as reported by Silva & Gonçalves (2017) [79], who treated Nile tilapia fillets (*Oreochromis niloticus*) with ozonated water (0.5, 1.0 and 1.5 mg/L for 0 to 15 min) and observed a reduction of about 3 and 4 log units of total aerobic mesophiles in the whole tilapia-surface at an ozone concentration of 1 and 1.5 mg/L and an exposure time of 10 and 15 min. Regarding tilapia fillets (*Oreochromis niloticus*), lower microbial loads reductions were observed, with both concentrations of 1.0 and 1.5 mg/L and exposure times of 10 and 15 min having very similar effects. Nevertheless, the inactivation rates decreased by increasing the exposure time, with the authors attributing this effect to the decreasing oxidizing effect of ozone due to its instability and consequent dissociation, releasing oxygen to water. Lipid oxidation increased with exposure time and ozone concentration, which can also be explained by the dissociation of ozone and release of oxygen, promoting lipid oxidation, increasing the formation of peroxides and/or products resulting from their decomposition. The proposed mechanism of lipid-enhanced oxidation by ozone relies on the generation of free O_2_^-^ and HO^.^ radicals that have a high oxidation-reduction potential. Generation of these radicals in water (H_2_O^−^ can be formed by reacting with O_2_^−^ and HO) enhances lipid peroxidation and protein denaturation [80]; thus, both concentration and exposition time are to be carefully controlled to avoid undesirable changes in the fish surface. Some examples of the effects of ozone dipping and spraying are displayed in Table 4.

Ozone-washing can also be used as a tool to remove muddy-flavors (such as those derived from geosmin), as reported by Zhang et al. (2016) [88], who was able to remove 69.2% of geosmin after an ozuone-flotation treatment for 15 min, against 54.3% for conventional ozone water washing for the same treatment time.

##### Ozonized Icing-Systems

The ozone-icing process consists in preparing ice flakes with ozone that will be directly in contact with the fish surface, allowing fresh fish to be kept under chilling conditions near 0 °C, in a similar process described previously for organic acid icing-systems.

For example, Campos, Losada, Rodríguez, Aubourg, and Barros-Velázquez (2006) [89] evaluated the effects of ozonated slurry ice (−1.5 °C), and ozone concentration of 0.2 mg/L to preserve turbot (*Psetta maxima*) stored at 2 °C for 35 days, and observed that lipid hydrolysis and oxidation slowed down in the presence of ozone in the slurry ice, in addition to the lower microbial loads in fish individuals (nevertheless, parameters such as nucleotide degradation and TMA-N were not influenced by the presence of ozone). The authors also reported a sensorial shelf-life for ozonated slurry ice of 14 days, compared to 7 days for control samples. Comparable results were obtained by Aubourg et al. (2006) [90] for megrim (*Lepidorhombus* *whiffiagonis*) preserved by ozonated slurry ice (at a concentration of 0.08 and 0.16 mg/L), with megrim individuals presenting improved sensorial attributes after 14 days of on-board storage when compared to un-ozonated slurry ice, showing that on-board ozonated slurry ice systems are an interesting approach to preserve fresh fish with minimal impact on fish quality and safety. Another study from Chen et al. (2016) [91] with bighead croaker (*Collichthys niveatus*) discovered that ozonated slurry-ice can retard the degradation of myofibrillar proteins and reduce the deterioration of fish microstructure. In addition, this methodology proved to have inhibitory effects on formation of TVB-N, peroxide index, and thiobarbituric acid reactive substances (TBARS) values.

### 2.4. Natural Extracts

The application of natural preservatives in foods has caught the attention of the general public. Foods containing natural preservatives will be chosen over those containing synthetic preservatives [2]. According to Gyawali & Ibrahim (2014) [92], natural preservatives should have a broad action against bacteria and fungi, be non-toxic, and be active in low concentrations, not change the color and flavor of foods, have no pharmaceutical applications, be clean-label, and cost-effective. Most natural preservatives are derived from microorganisms, animals, and plants [93].

#### 2.4.1. Extracts from Plants

Plants, especially their secondary metabolites, present a wide range of applications, especially in pharmaceutical and food sectors. These extracts usually show antimicrobial activity, attributed to polyphenolic compounds which come into contact with bacterial membranes and lead to their disruption and cell leakage, along with the formation of hydroperoxides [2]. In addition, these extracts may also present antioxidant activity, which may be interesting for fish preservation by means of slowing down lipid oxidation. Table 5 presents the effects of some plant extracts on fresh fish preservation.

Similar to organic acid dipping, the effects of plant extracts in fresh fish are dependent upon extract concentration, dipping time, and temperature, since excessive dipping can result in undesirable physicochemical and sensorial effects.

The origin of the extracts and extraction solvent may influence the evolution of fish degradation rates during refrigerated storage. For example, Yazgan et al. (2020) [100] evaluated the feasibility of water and ethanol extracts of propolis (0.4 and 0.8%) in sardine (*Sardine aurita*) and observed microbial load inhibition for both extracts, which was more pronounced for ethanolic extracts. The authors also reported that, despite ethanolic extracts providing the best sensorial scores, chemical parameters such as TBARS and peroxide values were lower for water extracts when compared to ethanolic extracts, showing that the extract composition (influenced by the extraction solvent) plays a significant role in antioxidant activity of the compounds extracted [101], and, consequently, on the feasibility of the extracts to preserve fresh fish. The fact that the sardine samples were vacuum-packed also inhibited some oxygen-dependent reactions.

In this sense, the addition of natural extracts can even be combined with other hurdles to microbial development to further improve the shelf-life of fresh fish. Houicher et al. (2015) [102] combined VP with ethanolic extracts of mint and artemisia on sardine (*Sardina pilchardus*) kept under refrigeration for 21 days. Both extracts, at 1% concentration, yielded lower values of histamine, tyramine, and cadaverine, while mint extracts were more effective to decrease biogenic amines production. Nevertheless, artemisia extracts were more effective to inhibit histamine-forming bacteria.

The application of natural extracts in icing-systems was assessed by Miranda et al. (2018) [103], who evaluated the combined effect of ethanolic quinoa extracts (at a concentration of 0.05 and 0.20%) and icing-system to preserve Atlantic chub mackerel (*Scomber colias*). The results showed that lipid hydrolysis was lower during the 13 days of storage, which correlated well with lipolytic bacteria growth slow down. In addition, the peroxide, TBARS and fluorescent compound values observed for fish individuals in the icing-system with 0.20% quinoa extracts were lower than those without quinoa extracts. These results were attributed to the phenolic compounds and other hydrophobic compounds with antimicrobial activity.

Indeed, the use of natural extracts in combination with other hurdles (such as MAP and vacuum packaging, refrigeration, etc.) can enhance the shelf-life of fresh fish even further, as an additional hurdle (provided by the natural extracts) is being added to the system. The combination of multiple natural extracts is also reported in the literature, although it must be carefully studied to avoid loss of fresh fish sensorial attributes [104]. Table 6 summarizes some examples of the use of the multi-hurdle concept for fish preservation, and the main effects on the fish products.

In a circular economy approach, the use of plant-based agri-food wastes with antimicrobial activity may be an interesting approach to valorize these wastes, allowing reduction of food waste. For example, Shinde et al. (2015) [109] reported a 2-fold shelf-life of Indian mackerel (*Rastriliger kanagurta*) dipped in 0.5 and 1% of pomegranate peel extract, which also resulted in lower values of TMA-N and TVB-N and lower lipid hydrolysis for both concentrations. As expected, higher concentrations of extract allowed improved control of microbial growth. Pomegranate peels are very rich in flavonoids and tannins that are known to have high antioxidant activity, along with other agri-food wastes, such as potato and apple peels, rice bran, and grape seeds, among others, which may be an interesting research topic to evaluate in coming years.

Plant-based extracts can be combined with other natural compounds, such as bacteriocins, which will provide an amplified antimicrobial effect and improved physicochemical stability of fresh fish. In this sense, Gao et al. (2014) [97] combined rosemary extracts with nisin on pompano fish (*Trachinotus ovatus*) and observed that the combination of extracts resulted in lower values of TBARS, peroxide indexes, TVB-N, TMA-N and improved sensorial and texture parameters, along with microbial development slow down.

#### 2.4.2. Extracts from Algae

Algae have received a lot of attention lately due to their abundance, being natural sources of terpenes, phlorotannins, phenolic compounds, fatty acids, steroids, halogenated ketones and alkanes, acrylic acid, polysaccharides, and cyclic polysulphides that have potent antioxidant and antimicrobial capacity [2]. Considering this, Wang et al. (2009) [110] reported that phlorotannins can inhibit oxidative phosphorylation and binding to bacterial membranes, proteins, and enzymes, leading to cell lysis. In addition, Amorim et al. (2012) [111] suggested that some sulphated polysaccharides from algae may bind to cell wall compounds and cytoplasmic membrane and penetrate within the cell and bind to DNA, which will increase cytoplasmatic membrane permeability, thus slowing down microbial development. As an example, Miranda et al. (2018) [103] included ethanolic extracts from *Bifurcaria bifurcata* to evaluate their potential to extend the shelf-life of megrim (*Lepidorhombus whiffiagonis*). During the 14 days of storage, it was observed that indigenous microorganisms were inhibited by the algae extracts, and TMA-N values and lipid hydrolysis were lower for fish samples with algae extracts. Oucif et al. (2018) [112] combined ethanolic-water extracts of the algae *Cystoseira compressa* with an icing system to preserve horse mackerel (*Trachurus trachurus*) and observed that microbial development slowed down during 11 days of storage. The addition of algae extracts also resulted in lower lipid hydrolysis rates, along with lower values of TMA-N and reduced lipid oxidation (primary, secondary, and tertiary) compared to control samples (where no extract was added).

Other examples considered the addition of algae extracts to preserve fish, namely tilapia (*Oreochromis niloticus*) [113], tuna (*Euthynnus affinis*) [114], Atlantic salmon (*Salmo salar*) [115], Atlantic chub mackerel (*Scomber colias*) [103], among others.

#### 2.4.3. Latic Acid Bacteria and Bacteriocins

According to Huss et al. (1995) [116], candidate protective cultures for fish preservation must fulfil four essential criteria, as follows: (1) present no threats to health; (2) have the ability to grow and compete with other cultures under refrigeration conditions (5 °C); (3) must present a consistent and continuous antimicrobial activity; (4) must not spoil fish (by means of off-flavors and odor production, slime, etc.).

Bacteriocins are usually produced by LAB in order to create a more competitive environment for nutrient uptake, i.e., while bacteriocins inhibit other microbial consortia proliferation, LAB take advantage of such inhibition and can uptake the available nutrients [117]. Several studies have demonstrated the effectiveness of bacteriocins (peptides containing between 30 and 60 amino acids), especially those produced by LAB against fish pathogens and degradative microorganisms [2]. Nisin, lacticin, pediocins, and reuterin (an organic compound derived from D-ribose) are, by far, the most studied bacteriocins for preserving fresh fish. Nevertheless, this strategy relies on direct application of LAB cultures by fish dipping/spraying, or by application of the isolated bacteriocins [2].

In this sense, Cao, Liu, Chen, Yang, and Li, (2015) [118] evaluated the effects of *Lactobacillus plantarum* 1.19 dipping of tilapia (*Oreochromis niloticus*) fillets and observed a lower increase in K-value (freshness index of fish flesh, whereas high K-values are related with fish spoilage) and total aerobic mesophiles during refrigerated storage (4 °C), as well as a better sensorial score when compared to undipped samples. In another study, Anacarso et al. (2014) [119] sprayed fresh Atlantic salmon (*Salmo salar*) with *Lactobacillus pentosus* 39 and observed its activity against *Aeromonas hydrophila* (a Gram-negative foodborne pathogenic bacteria that is quite resistant to sanitizing agents and able to thoroughly reproduce under refrigeration conditions) and *L. monocytogenes*, suggesting that, under refrigeration conditions, *Lact. pentosus* 39 was able to reduce the counts of both aforementioned microorganisms, in addition to the total psychrophilic bacteria. Although, the success of this technique requires a good control of the cold-chain, as a simulated breakage of the cold-chain (for 12 h) resulted in the development of both pathogenic microorganisms.

When it comes to the direct application of bacteriocins, Sarika and colleagues [120] studied the effects of three different concentrations of enterocin CD1 (0.1, 1 and 10% *v*/*v*) on the preservation of reef cod fish (*Epinephelus diacanthus*) and reported a 2-log units reduction of total aerobic microorganisms for the highest concentration of enterocin CD1 after 28 days of storage at 4 °C, with the authors also reporting that this bacteriocin performed better than the conventional sodium benzoate to extend the shelf-life of cod. The regulatory status on the use of LAB and bacteriocins for food preservation must be carefully considered. When it comes to regulatory status, nisin is the only bacteriocin licensed as a biopreservative. To overcome this limitation, in situ production by lactic acid bacteria is a common approach and several bacteriocinogenic cultures are commercially available. To be used in food production, LAB species must be granted the FDA GRAS designation or be included in the European Food Safety Authority’s (EFSA’s) list of qualified presumption of safety (QPS) recommended biological agents [121,122].

Further research is needed to evaluate the impact of bacteriocins and lactic acid bacteria on fish quality and safety, as well as to meet regulatory requirements to ensure their safe application in the food industry.

## 3. Conclusions

Refrigeration and freezing are common methods used to preserve fresh fish, which is perishable due to its rich nutritional composition and physicochemical characteristics. However, these low temperature-based technologies cause some detrimental quality changes, such as off-odors, off-flavors, rancidity, and loss of texture; therefore, several technologies have been introduced to improve the overall preservation of the product.

Vacuum and modified atmosphere packaging technologies are characterized by the manipulation of the atmosphere of the package, offering an additional hurdle to the physical barrier protecting the fishery products. This allows the slowdown of microbial deterioration and the rate of degradation reactions and, consequently, an increase in the shelf-life. Overall, increases in the shelf-life of fresh fish by MAP of 2/3 days and 4/5 days, when compared with VP and air packages, respectively, along with better sensorial quality have been reported.

By adding active compounds with antimicrobial and/or antioxidant capacities to synthetic or natural packaging films, active packaging technology has a positive impact on the shelf-life and quality characteristics of fresh fish. As in active packaging, chemical additives (like organic acids and ozone) and natural extracts make it possible to slow down microbial proliferation and decrease TVB-N and TMA-N values, both important parameters related to the shelf-life and sensorial quality of fresh fish.

Despite the advantages of the use of chemical-based strategies for fresh fish preservation here presented and discussed, both regulatory and consumer approval challenges must be overcome until real commercial applications can be safely used in the industry. Moreover, an intense validation procedure must be performed to fully elucidate and convince regulatory authorities on the safe use of natural extracts as food preservatives, alongside with raising consumer awareness of the advantages of such procedures in the food industry. Nevertheless, even though some of the presented preservatives exemplified here come from natural sources, their toxicity is yet to be evaluated, as well as the interactions that such compounds can have either with the fish products or packaging.

In conclusion, these chemical-based strategies are proven to successfully impact fresh fish preservation and the interest in their application will certainly warrant new developments in research in the future.

## Figures and Tables

**Table 1:** Literature regarding the effects of air-packaging, vacuum packaging (VP), and modified atmosphere packaging (MAP) on fresh fish.

Species (*Scientific Name*)	Storage Conditions (Temperature, Duration)	Major Results	Reference
Atlantic Salmon (*Salmo salar*)	1.2 °C, 25 days Packaging: MAP (60% CO_2_ and 40% N_2_) and VP	MAP (15/18 days) extended the shelf-life 7 days compared to VP samples (8/11 days). Negative odors and liquid losses were detected earlier for VP. Lower firmness and higher color intensity in MAP samples.	[29]
Chub mackerel (*Scomber colias japonicus*)	3 °C and 6 °C, 15 days Packaging: MAP (50% CO_2_ and 50% N_2_), air and VP	Lower pH values on MAP and VP samples. Longer shelf-life in MAP (12–10 days), followed by VP (10–8 days) and air (8–7 days) at 3 and 6 °C, respectively; Faster growth and higher microbial load for air samples.	[28]
Chub mackerel (*Scomber japonicus*)	1 °C, 14 days Packaging: air and VP	VP reduced TMA content but was ineffective to reduce biogenic amine contents.	[30]
Cod (*Gadus morhua*)	2 °C, 15 days Packaging: MAP (60% CO_2_ and 40% N_2_) and VP	Shelf-life of 7 days for VP and 9 days for MAP samples.	[26]
Common carp (*Cyprinus carpio*)	3 °C, 15 days Packaging: MAP (1: 40% CO_2_ and 60% N_2_ 2: 100% CO_2_)	MAP2 slowed total viable microorganisms’ growth, presented significant lower counts of Enterobacteriaceae, lower pH value and no sensory changes were detected throughout the storage period. Higher values of TVB-N were observed for MAP1.	[27]
Common carp (*Cyprinus carpio*) and rainbow trout (*Oncorhynchus mykiss*)	3 °C, 14 days Packaging: MAP (1: 60% CO_2_ and 40% N_2;_ 2: 40% CO_2_ and 60% N_2_) and VP	Higher counts of Enterobacteriaceae for VP samples, followed by MAP2 and finally MAP1 in both fish species.	[31]
Grass Carp (*Ctenopharyngodon idellus*)	4 °C, 8 days (air), 16 days (VP) or 24 days (MAP) Packaging: MAP (75% CO_2_ and 25% N_2_), air and VP	Doubled and tripled sensorial shelf-life for VP (16 days) and MAP (24 days), respectively. Lower pH and improved sensorial parameters for VP and MAP. Significantly higher TVB-N for air samples in the first 8 days. Higher levels of tyramine for VP and MAP, and putrescine and cadaverine for air samples.	[32]
Meagre (*Argyrosomus regius*)	4 °C, 8 days (air) or 13 days (VP) Packaging: air and VP	Increased shelf life by approx. 4 days for VP (~10 days). Lightness and hardness increased over time regardless of the type of packaging. Significantly less microbial growth on VP samples.	[11]
	4 °C, 15 days Packaging: MAP (40% CO_2_, 30% N_2_ and 30% O_2_), air and VP	Microbial loads were significantly lower under VP. Color was not affected by any packaging method.	[23]
Rainbow trout (*Oncorhynchus mykiss*)	4 °C, 22 days Packaging: MAP (80% CO_2_ and 20% N_2_), air and VP	MAP reduced total production of ammonia, TVB-N and cadaverine. MAP enhanced the shelf-life at least twice since total mesophilic count and psychotropic microorganisms reached the upper limit of 7 log CFU g^−1^ on the 11th day (air—5th day; VP—7th day).	[21]
Red Drum (*Sciaenops ocellatus*)	4 °C, 29 days Packaging: MAP (50% CO_2_ and 50% N_2_) and VP	Putrescine and cadaverine were the prevalent amines and had higher counts on VP. At the end of the storage VP samples retained a slightly better appearance than MAP samples, with a firmer texture but had a stronger odor.	[6]
Saithe (*Pollachius virens*)	4 °C, 13 days Packaging: MAP ● *high CO_2_/low N_2_*: 67.2 ± 0.2% CO_2_, 32.8 ± 0.2% N_2_ and 0.0 ± 0.0% O_2_ ● *low CO_2_/high N_2_*: 31.8 ± 0.2% CO_2_, 68.2 ± 0.3% N_2_ and 0.1 ± 0.1% O_2_ ● *high CO_2_/low O_2_*: 66.4 ± 0.4% CO_2_, 32.2 ± 0.0% O_2_ and 1.3 ± 0.3% N_2_ ● *low CO_2_/high O_2_*: 31.3 ± 0.2% CO_2_, 66.0 ± 0.1% O_2_ and 2.7 ± 0.3% N_2_ ● and VP	All MAP conditions had the same shelf-life of 13 days, 3 days longer than VP samples (10 days). Lower muscle pH was observed in packages balanced with O_2_ compared to those balanced with N_2_. Differences were found between off-odors produced in MAP with mix of CO_2_ and O_2_ (butter-like) and a mix with CO_2_ and N_2_ (ammonium-like). Drip loss was higher in “*high CO2/low N2*” MAP. Higher cadaverine formation in packages balanced with N_2_.	[24]
Sardine (*Sardina pilchardus*)	3 °C, 15 days Packaging: MAP (50% CO_2_ and 50% N_2_) and air	Longer shelf-life for MAP (9 days), followed by VP (7 days) and air (5 days). Higher concentration of ammonia and a significant increase of pH in air samples.	[33]
Sea bass (*Dicentrarchus labrax*)	4 °C, 21 days Packaging: MAP (1: 40% CO_2_, 50% N_2_ and 10% O_2_ 2: 60% CO_2_, 30% N_2_ and 10% O_2_) and air	MAP1 extended shelf-life by 3 days (8/9 days) while MAP2 extended it by 7/8 days (13 days) based on sensory analysis. Lower TVB-N and TMA-N values for MAP2 samples.	[9]
Silver carp (*Hypophthalmichthys molitrix*)	4 °C, 14 days Packaging: air and VP (30-50 kPa)	Significant decrease of microbial growth with VP; Lower pH and TVB-N for VP samples; Better sensory quality and increased shelf-life (by 3 days) for 30 kPa VP samples.	[20]
Sutchi catfish (*Pangasius hypophthalmus*)	4 °C, 21 days Packaging: MAP (1: 50% CO_2_ and 50% N_2_; 2: 50% CO_2_ and 50% O_2_), air and VP	MAP with O_2_ significantly extended the lag phase compared to the MAP without O_2_. Shelf-life was extended by 3, 5, and 7 days with VP (10 days), MAP 1 (12 days) and MAP2 (14 days), respectively, in comparison to air samples.	[25]
Swordfisfh (*Xiphias gladius*)	4 °C, 18 days Packaging: MAP (50% CO_2_, 45% N_2_ and 5% O_2_) and air	Microbial and sensorial shelf-life extension by 5/6 days on MAP samples (12/13 days) compared to air samples (6/8 days). Lower values of TMA-N for MAP samples.	[10]
Tropical yellowfin tuna (*Thunnus albacares*)	0 °C for air, 4 °C in the first week then 8 °C for VP and MAP, 13 days Packaging: MAP (70% CO_2_ and 30% O_2_), air and VP	No extension of shelf-life was provided by VP and MAP (13 days). Similar bacterial evolution. Very low levels of TVB-N and no differences between treatments. TMA-N increased for MAP and VP samples but not for air samples. VP and MAP presented a slight discoloration and MAP samples were less firm.	[34]
Yellow grouper (*Epinephelus awoara*)	0 °C, 15 days Packaging: VP	TVB-N and TMA-N significantly increased over time. Significant variations for hardness, gumminess, and chewiness values with storage time. Evolution of color to grey-blue tones and reduction in color intensity and purity.	[8]

TVB-N: Total volatile basic-nitrogen; TMA-N: Trimethylamine-nitrogen.

**Table 2:** Natural active compounds incorporated into natural/synthetic polymers used for fish and fishery products.

Fish Specie (*Scientific Name*)	Polymers	Active Compounds	Main Results	Reference
Fish *	Poly (butylene adipate co-terephthalate)—PBAT	Oregano (*Origanum vulgare*) essential oil (OEO)	A high antioxidant action and antimicrobial effect due to the lower counts of coliforms, *Staphylococcus aureus* and psychrotrophic microorganisms.	[45]
Flounder (*Paralichthys orbignyanus*)	Agar	Fish protein hydrolysate (PH) or clove essential oil (CEO)	Improvement of the biochemical (TVB-N and pH values, etc.) and microbiological (H_2_S-producing bacteria, etc.) parameters of chilled flounder fillets and, thus, increasing the shelf-life.	[43]
Hake (*Merluccius capensis*)	Agar	Green tea extract (*Camellia sinensis* L.) and probiotic bacteria (*Lactobacillus paracasei* L26 and *Bifidobacterium lactis* B94)	Green tea films effectively reduced microbial growth and some spoilage indicators such as TVB-N, TMA-N, and pH value, in hake. Probiotic films have been able to extend the shelf-life of hake and transmit some probiotic bacteria to fish.	[46]
Rainbow trout (*Oncorhynchus mykiss*)	Chitosan-gelatin	Ethanolic red grape seed extract (GSE) and *Ziziphora clinopodioides* essential oil (ZEO)	Reduction of lipid oxidation and bacterial growth, increasing the shelf life of rainbow trout at refrigerated storage.	[44]
Salmon (*Salmo Solar*)	Low Density Polyethylene (LDPE)	Natural tocopherols (commercial names: NUTRABIOL^®®^ T90 and TOCOBIOL^®®^—PV)	Antioxidant effectiveness, through the reduction/inhibition of the lipid oxidation of salmon during storage period, by up to 40%.	[42]
	Cassava starch	Extract of microalgae *Heterochlorella luteoviridis*	A reduction in lipid oxidation and moisture loss.	[47]

TVB-N: Total volatile basic-nitrogen; TMA-N: Trimethylamine-nitrogen. * The authors did not mention the scientific name of the species.

**Table 3:** Literature regarding the effects of organic acids dipping and spraying on fresh fish.

Specie (*Scientific Name*)	Organic Acid or Salt (Concentration, Dipping Time)	Storage Donditions	Results	Reference
Bigeye trevally (*Caranx sexfasciatus*)	Lactic, acetic, and citric acids (all at 2%, 30 min)	5 °C, vacuum packaging, for 7 days	Elimination of pathogenic *Escherichia coli* and *Listeria monocytogenes*. Total aerobic mesophiles development slowed down. Minor color changes because of the dipping process.	[60]
Black pomfret (*Formio niger*)	Sodium acetate (2.5%, 5 min)	4 °C, air packed, for 7 days	Improved moisture retention, tenderness, and higher water holding capacity, lower drip loss, and total TMA-N and TVB-N values in dipped fish samples compared to undipped fish.	[66]
Bolti fish (*Oreochromis niloticus*)	Acetic and citric acid (1 and 3%, respectively, 5 min) and a mixture of both	4 °C, air packed, for 12 days	Slower microbial proliferation, along with catalase and protease activity decrease in dipped fish, especially when the mixture of both acids was used.	[64]
Catifish fillets (*)	Lactic acid (1.70 and 2.55%, 10 min)	2 and 7 °C, air packed, for 3 and 6 days, respectively	Shelf-life extension up to 6 days for 2.55% dipped samples stored at both temperatures. Sensorial panel did not consistently distinguish dipped and undipped samples.	[67]
	Lactic acid (2%, 5 min) (3%, 1 and 5 min)	4 and 10 °C, air packed, for 9 days	Dipped samples presented less than 2 log units of Gram-negative bacteria by the 9th day of storage. Higher concentrations and dipping times slowed down microbial development.	[56]
Catifish fillets (*Ictalurus punctatus*)	Acetic, citric, hydrochloric, lactic, malic, and tartaric acids (2%, 10 min)	4 °C, air packed, for 8 days	Reduced microbial proliferation (total aerobic mesophiles, total coliforms, and *L. monocytogenes*) on acid treated samples. Significant color changes in catfish fillets after dipping (malic acid had the smallest impact on lightness while yellowness was less impacted by hydrochloric acid).	[68]
Chub mackerel (*Scomber japonicus*)	Lactic acid (0, 2 and 4%, 30 min)	4 °C, vacuum packed, for 12 days	Shelf-life extension of lactic acid dipped fillets. Improved control of TMA-N and TVB-N production.	[69]
Nile tilapia (*Oreochromis niloticus*)	Acetic and citric acid (1 and 3%, respectively, 5 min) and a mixture of both	4 °C, air packed, for 12 days	Reduction of thiobarbituric acid-reactive substances (TBARS) and TVB-N concentration. Improved WHC and lipid content for fish dipped in citric and acetic acid compared to undipped fish.	[63]
	Acetic acid (1%, 2 min)	2 °C, air and modified atmosphere (80% CO_2_ and 20% N_2_) packaging, for 21 days	Microbiological shelf-life extension for modified atmosphere packaged dipped fish samples, improvement of TVB-N and TBARS values, and good overall acceptability after 21 days of storage.	[70]
Pearl spot (*Etroplus suratensis*)	Sodium acetate and potassium sorbate (both at 2%, 30 min)	1–2 °C, air and vacuum packed, for 18 days	Microbiological and sensorial shelf-life extension of vacuum packaged pearl spot when combined with salts (16 days, compared to 7 days for control samples), with improved sensorial properties and reduced TMA-N and TVB-N compared to untreated samples (without salts).	[57]
Salmon (*Salmo salar*)	Sodium acetate, sodium citrate and sodium lactate (2.5%, 10 min)	1 °C, air packed, for 15 days	Microbial development inhibited by dipping treatment, with sodium citrate showing the best results. Both lipid oxidation and TBARS values were delayed.	[62]
			Shelf-life extension of 12 days for sodium lactate and sodium citrate, and 15 days for sodium acetate. Reduction of k-value, hypoxanthine, TVB-N, TMA-N values, and improved sensorial attributes in dipped salmon fillets.	[71]
Sardine (*Sardina pilchardus*)	Lactic acid (5%, 2 min)	4 °C, air packed, for 7 days	Lower total aerobic mesophiles and *Pseudomonas* spp. counts for lactic-acid dipped fish samples. Improved odor, appearance, and aroma compared to undipped samples.	[72]
Seer fish (*Scomberomorus commerson*)	Sodium acetate (2%, 10 min)	1–2 °C, packed in air permeable ethylene vinyl alcohol, for 24 days	Shelf-life extension of dipped fish samples (21 days) compared to undipped samples (12 days). Extended lag phase of microbial development in dipped samples. Reduced lipid oxidation and nucleotide breakdown inhibition in dipped fish.	[73]
Silver carp (*Hypophthalmichthys molitrix*)	Acetic and ascorbic acid (1 and 2%, respectively, sprayed) and a mixture of both	4 °C, air packed, for 9 days	Lower microbial loads, pH, and peroxide values in fish fillets sprayed with the combination of both organic acids, along with improved sensorial characteristics.	[74]

* The authors did not mention the scientific name of the species.

**Table 4:** Literature regarding the effects of dissolved ozone dipping and spraying on fresh fish.

Specie (*Scientific Name*)	Concentration, Dipping Time, Water Temperature	Storage Conditions	Results	Reference
Catfish (*Ictalurus punctatus*)	5 and 10 mg/L, 10 min, 20 °C	4 °C, air packed, for 12 days	Total psychrophiles and coliform loads reduction after fish dipping, minor impact on microbial evolution during storage. TBARS values remained unchanged for 12 days.	[81]
Cod (*Merluccius merluccius*)	3.5 mg/L, 3 cycles of 5 min and 4.7 mg/L, 4 cycles of 10 min, **	2 °C (passive refrigeration), air packed, for 12 days	Microbial development inhibition. Lower TVB-N and TMA-N values, along with higher lipid hydrolysis and TBARS values compared with undipped samples.	[82]
Nile tilapia (*Oreochromis niloticus*)	4.0 mg/L, 30 min, **	Ice storage (replaced every 24 h), air packed, for 18 days	Microbiological shelf-life extension for ozonated samples, along with lower TVB-N and higher TBARS values. No sensorial differences between unozonized and ozonized samples.	[80]
Rainbow trout (*Onchorynchous mykiss*)	0.6 and 0.4 mg/mL, for 60 and 90 min respectively), 5 °C	4 °C, vacuum packed, for 15 days	Microbial development retarded by ozone dipping, although with minor differences between ozone concentrations and dipping time. TVB-N values for dipped samples were considerably lower.	[83]
Red mullet (*Mullus surmuletus*)	0.3 mg/L, 10 min, 5 °C	1 °C, MAP (50% CO_2_ and 50% N_2_), for 24 days	Lower microbial loads, TVB-N and TMA-N levels compared to unozonized samples under MAP. Similar peroxide values and sensorial acceptability.	[84]
Salmon (*Salmo salar*)	1 and 1.5 mg/L, 1–3 spray nozzles, **	4 °C, air packed, for 10 days	Inhibitory effect against *Listeria innocua* for 6 days. Lower TBARS and propanal values for ozone-spayed fish, especially at 1.5 mg/L. Number of spray nozzles showed minor impacts on the aforementioned parameters.	[85]
Scaldfish (*Arnoglossus laterna*)	8 mg/L, 6 cycles of 5 min, **	2 °C, air packed, for 12 days	Shelf-life extension of 1 week by microbial development inhibition in ozonated samples. Lower TVB-N lipid hydrolysis, along with higher TMA-N and TBARS values compared to undipped fish.	[82]
Tilapia (*Oreochromis niloticus* x *Oreochromis aureus*)	0.1 mg/L, 1 h, 20 °C	0–5 °C, air packed, for 30 days	Microbiological and sensorial shelf-life extension of ozonized samples compared to control, improved freshness, especially for those kept at 0 °C. Lower TVB-N, TBA, and values for ozonized tilapia muscles along with lower scores for odor and taste. No effects on texture.	[86]
Trout *	0.1 mg/L, 2 h, **	5 °C, air packed, for 9 days	Shelf-life extension of ozonized trout from 4 to 6 days compared to control samples, along with lower values of TVB-N and peroxides’ index. No major differences in protein content for ozonized samples compared to controls.	[87]

TVB-N: Total volatile basic-nitrogen; TMA-N: Trimethylamine-nitrogen; TBARS: Thiobarbituric acid reactive substances; TBA: Thiobarbituric acid. * The authors did not mention the scientific name of the species. ** The authors did not mention the water temperature.

**Table 5:** Examples of the application of natural extracts from plants to fresh fish and their effects on fish quality indicators.

Species (*Scientific Name*)	Plant or Extract, Concentration, Dipping Time, Temperature	Storage Conditions	Results	Reference
Black bream (*Acanthopagrus butcheri*)	Kakadu plum, 0.05–0.2%, 6 h, **	4 °C, air packed, 15 days	Both leaf and fruit extracts slowed down microbial development, especially at higher concentration.	[94]
Crucian carp (*Carassius auratus*)	Rosemary, 0.2%, 2 min, 4 °C	4 °C, air packed, 20 days	Sensorial shelf-life extension (2-fold) of fish samples dipped in rosemary extract, validated by microbiology results, along with pH, TVB-N, and k-value increase.	[95]
Nile tilapia (*Oreochromis niloticus*)	Moringa, 1–4%, ice incorporation	5 °C, ***, 12 days	Microbial growth delayed with increasing moringa extract concentration. Lower peroxides, TBARS, and TVB-N values compared to control samples (with no extracts).	[96]
Pompano (*Trachinotus ovatus*)	Rosemary, 0.2%, 30 min, 4 °C	4 °C, air packed, 15 days	Lower microbial loads and k-values during storage, along with lower TMA-N and TVB-N values compared to control. No effects on TBARS values and peroxide index.	[97]
Rainbow trout (*Oncorhynchus mykiss*)	Cumin and wild mint, 3–6%, 30 min, **	4 °C, air packed, 18 days	Wild mint extract provided the best antimicrobial and antioxidant activities, resulting in lower peroxide, TBARS, TVB-N, and TMA-N values, along with the best sensorial scores.	[98]
Silver carp (*Hypophthalmichthys molitrix*)	Few-flowered garlic, 2.0–4-0%, 30 min, **	4 °C, air packed, 15 days	Shelf-life extension of fish treated with garlic extracts of up to 15 days. Lipid oxidation rates slowed down. Improved sensorial attributes.	[98]
Zander (*Lucioperca lucioperca*)	Green tea, 1%, 10 min, **	4 °C, air packed, 15 days	Lower microbial counts, TVB-N and TBARS values, improved organoleptic scores. Lower content in histamine, cadaverine, and putrescine.	[99]

TVB-N: Total volatile basic-nitrogen; TMA-N: Trimethylamine-nitrogen; TBARS: Thiobarbituric acid reactive substances. ** The authors did not mention the dipping temperature. *** Fish samples were in direct contact with the ice.

**Table 6:** Examples on the application of single and combined natural extracts from plants with other hurdle techniques on fresh fish and their effects on fish quality indicators.

Species (*Scientific Name*)	Plant Extract, Concentration, Dipping Time, Temperature	Storage Conditions	Results	Reference
Sardine (*Sardine aurita*)	Propolis, 0.4–0.8%, 4 min, *	3 °C, vacuum packed, 15 days	Higher doses of water and ethanolic extracts improved sardine shelf-life and resulted in lower TVB-N and TBARS. Ethanolic extracts provided the best sensorial scores.	[100]
Sardine (*Sardinella pilchardus*)	Rosemary, 1–2%, 2 min, *	4 °C, vacuum packed, 20 days	Sensorial analyses scores were better for samples treated with 1% of rosemary extracts, despite the lower TVB-N, peroxide index, and TBARS values for 2% concentration.	[105]
Yellow corvina (*Larimichthys polyactis*)	Bayberry leaf, 0.2%, 1 h, 4 °C	4 °C, air and vacuum packed, 16 days	Microbial growth slow down. Lower values of TVB-N and TBARS values. Improved sensorial scores. No impact on fish color.	[106]
Sea bream (*Sparus auratus*)	Grapefruit seed extract (GFSE) and tymol and chitosan, 1500–6000 ppm(for GFSE and tymol) (1–4% chitosan), 60 s, *	4 °C, air and MAP (30:40:30 O_2_/CO_2_/N_2_ and 5:95 O_2_/CO_2_), 10 days	Inhibition of *Pseudomonas fluorescens* required a mininum active solution containing 2% of chitosan and 6000 ppm of GFSE and tymol. The use of MAP consisting of 5:95 O_2_/CO_2_, maintained the microbiological quality for up to 10 days, as opposed to undipped samples that spoiled in 3–4 days.	[104]
Common dolphin fish fillets (*Coryphaena hippurus*)	*H. strobilaceum*, 1%, 2 min, **	−1 °C and MAP (45% CO_2_, 50% N_2_, 5% O_2_), up to 18 days	Lipid oxidation was retarded and lower peroxide values and malondialdehyde content compared to control groups (placed in trays and not sealed). The use of *H. strobilaceum* allowed maintainance of the content of n-3 PUFAs	[107]
Pacific white shrimps (*Litopenaeus vannamei*)	Green tea, 0.1%, 15 min, 4 °C	4 °C and MAP (50% CO_2_, 5% O_2_, 45%, N_2_), up to 10 days	The combination of extracts and MAP allowed for better microbial growth control and lower melanosis formation compared to MAP alone.	[108]

TVB-N: Total volatile basic-nitrogen; TMA-N: Trimethylamine-nitrogen; TBARS: Thiobarbituric acid reactive substances; GFSE: Grapefruit seed extract. * The authors did not mention the scientific name of the species. ** The authors did not mention the dipping temperature.

## Data Availability

The data that support the findings of this study are available from the corresponding author, Jorge A. Saraiva, upon reasonable request.
